# An Uncommon Twist: Intussusception as a Rare Manifestation of Extramedullary Myeloma

**DOI:** 10.1155/cris/1872149

**Published:** 2026-09-15

**Authors:** Quinn E., Edwards Murphy A., Duggan W., DelaHarpe Golden P., Kavanagh D.

**Affiliations:** ^1^ Department of Surgery, Tallaght University Hospital, Tallaght, D24 NR0A, Dublin, Ireland, tallaghthospital.ie; ^2^ Department of Surgery, Royal College of Surgeons in Ireland, 123 St. Stephen’s Green, D02 YN77, Dublin, Ireland, rcsi.ie; ^3^ Department of Histopathology, Tallaght University Hospital, Tallaght, D24 NR0A, Dublin, Ireland, tallaghthospital.ie

**Keywords:** extramedullary plasmacytoma, intussusception, multiple myeloma, small bowel obstruction

## Abstract

We present the case of a 69‐year‐old female with relapsed multiple myeloma (MM) who presented acutely with small bowel obstruction secondary to a jejunal intussusception. Despite a trial of conservative management the obstruction did not resolve and surgical intervention was required. Laparoscopic‐assisted small bowel resection with primary anastomosis was performed. Histopathological examination revealed an extramedullary plasmacytoma with anaplastic features as the lead point of the intussusception. This case outlines the successful management of a rare but important oncological emergency and underscores the importance of considering extramedullary plasmacytoma in the differential diagnosis of bowel obstruction in patients with MM.

## 1. Introduction

Intussusception occurs when a segment of the intestine telescopes into an adjacent segment at a lead point, leading to intestinal obstruction [1]. While common in paediatric populations, adult intussusception is rare, accounting for 1%–5% of all cases of intestinal obstruction [2]. In adults, it is often secondary to an identifiable pathological lead point, with malignancies being a significant cause [3].

Diagnosing intussusception in adults can be particularly challenging due to its rarity and often vague, non‐specific presentation, typically with abdominal discomfort or intermittent pain [4]. While a detailed history and physical examination may raise clinical suspicion, imaging—especially abdominal computed tomography (CT)—plays a crucial role in confirming the diagnosis. Clinicians should therefore consider intussusception as a potential, albeit rare, cause of abdominal symptoms in adult patients. Where conservative measures fail, surgical intervention is frequently required to relieve the obstruction and resect or bypass an underlying pathological lead point, such as a malignancy. In cases where no lead point is identified, resection may not be necessary, and reduction of the intussuscepted segment may suffice [5].

Multiple myeloma (MM) is a haematologic malignancy of mature B cells, characterised by the presence of ≥10% clonal plasma cells in the bone marrow or a biopsy‐proven plasmacytoma. The diagnosis also requires evidence of end‐organ damage—such as hypercalcaemia, renal impairment, anaemia or bone lesions—attributable to the plasma cell disorder [6]. Extramedullary plasmacytomas represent plasma cell neoplasms occurring outside the bone marrow and can present as primary tumours or secondary to other plasma cell neoplasms, such as MM [7]. Secondary extramedullary plasmacytomas are noted in 7%–17% of MM cases and are associated with a poorer prognosis due to their aggressive behaviour [7, 8]. Gastrointestinal involvement by extramedullary plasmacytomas is rare, with the small intestine being an uncommon site [9]. Such involvement can lead to complications including intussusception and resultant bowel obstruction, requiring surgical intervention.

## 2. Case Presentation

A 69‐year‐old female presented with a 2‐day history of malaise, anorexia and three episodes of non‐bilious vomiting. Her background history included relapsed MM and chronic obstructive pulmonary disease. Her regular medications included long‐term oral corticosteroids (for suspected adrenal insufficiency), oral antihyperglycaemic agents, inhalers and antihypertensives. She denied abdominal pain, diarrhoea, rectal bleeding or urinary symptoms. Physical examination revealed a soft, non‐tender abdomen with normal bowel sounds. Chest auscultation demonstrated scattered wheeze and right basal crepitations.

**Figure 1:**
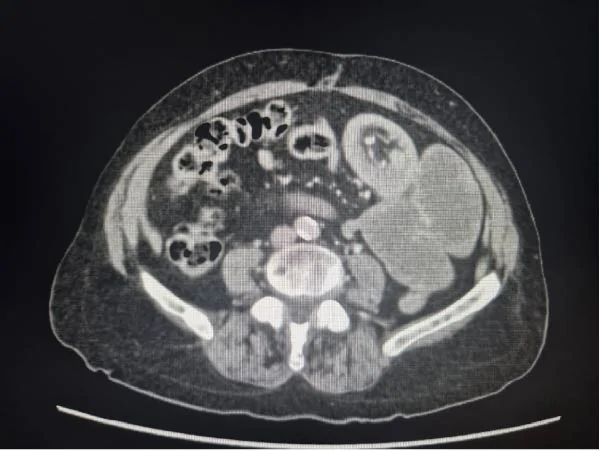
Axial slice of emergency CT abdomen pelvis demonstrating distended small bowel and two concentric hyperdense rings. Consistent with an intussusception.

**Figure 2:**
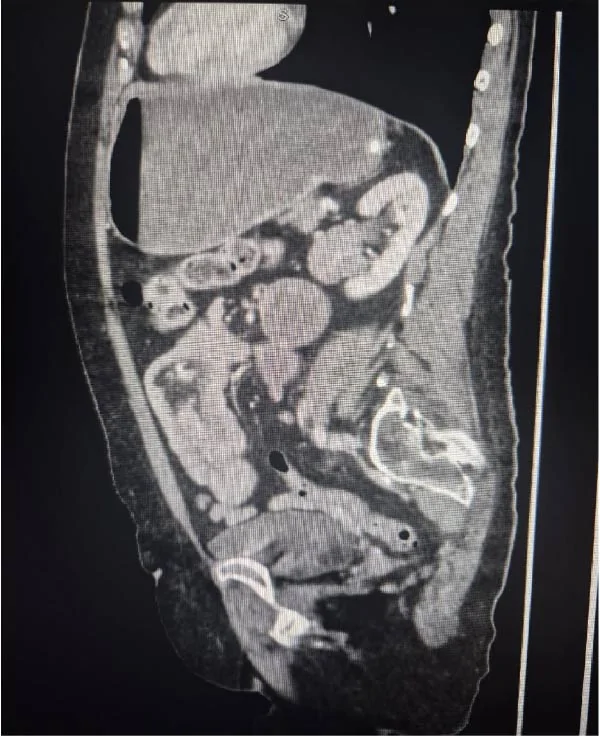
Sagittal view of emergency CT abdomen pelvis demonstrating intussusception as well as air fluid level with a distended stomach.

Laboratory investigations demonstrated leucocytosis (white cell count 18.2 × 10^9^/L), elevated C‐reactive protein (CRP) (173 mg/L), anaemia (haemoglobin 10.9 g/dL) and a lactate of 1.74 mmol/L. Renal function tests indicated an acute kidney injury superimposed on background chronic kidney disease. CT imaging of the abdomen and pelvis revealed a left‐sided jejunal intussusception with proximal small bowel dilatation (Figures 1 and 2), prompting an urgent surgical referral.

Initial management included nasogastric decompression, intravenous fluid resuscitation and total parenteral nutrition. High‐dose intravenous dexamethasone was prescribed under the direction of the haematology team with the dual aim of treating the underlying myeloma and reducing presumed peritumoral oedema in an attempt to relieve the obstruction. Despite three days of conservative treatment, there was no resolution of obstructive symptoms. Following careful discussion with the patient regarding the risks and benefits of operative intervention, informed consent was obtained for emergency laparoscopic exploration.

**Figure 3:**
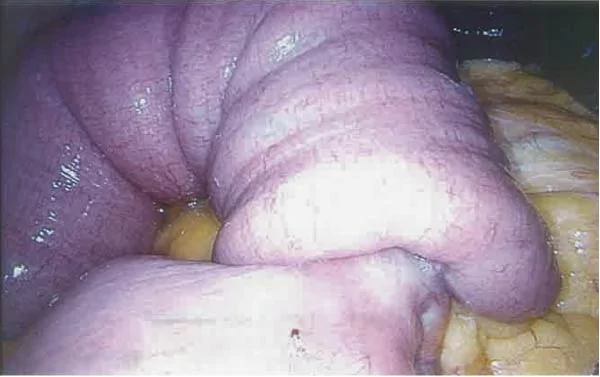
Intussusception extracorporeally at time of operation.

**Figure 4:**
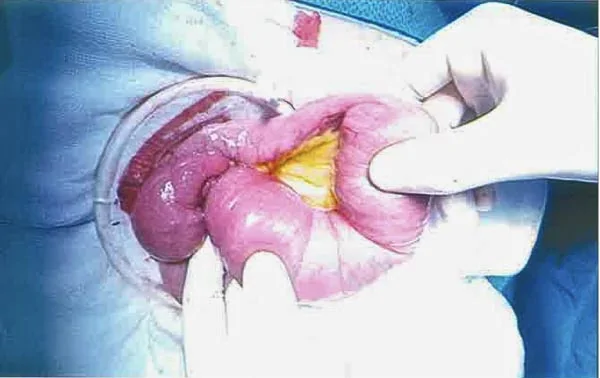
Intussuscipiens in both hands, delivered through wound protector. Collapsed bowel visible distally.

Intraoperatively, a laparoscopic small bowel survey identified a single, isolated intussusception with a collapsed small bowel distally. The affected segment was mobile and suitable for exteriorisation via the extension of the supraumbilical port site (Figures 3 and 4). A cicatrising, macroscopically suspicious lead‐point lesion was identified 40 cm distal to the duodenojejunal flexure. This segment of small bowel was resected with a corresponding mesenteric margin, and a side‐to‐side extracorporeal stapled anastomosis was fashioned. A completion small bowel survey distal to the anastomosis was performed to exclude further lesions, and laparoscopic haemostasis was confirmed.

**Figure 5:**
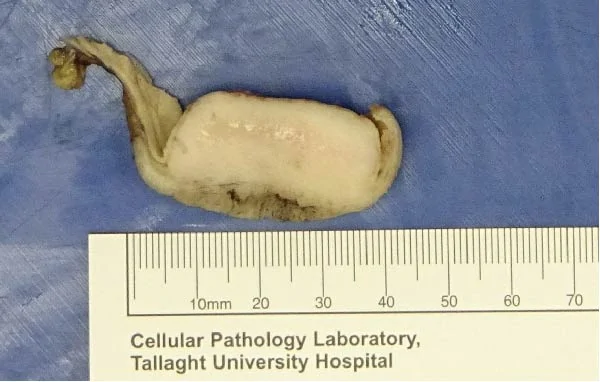
Macroscopic section of small bowel plasmocytic neoplasm.

**Figure 6:**
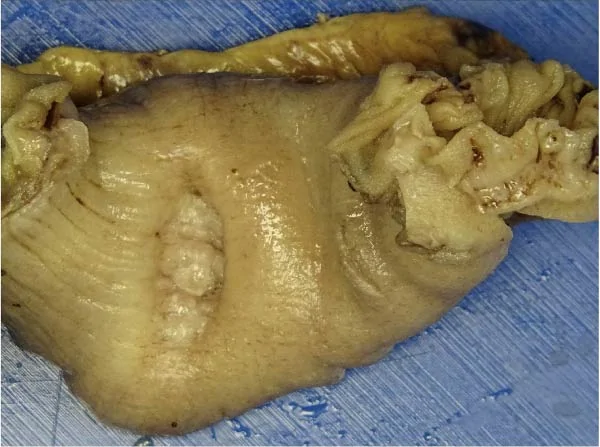
Macroscopic view of plasmocytic neoplasm involving the serosal layer of the small bowel.

**Figure 7:**
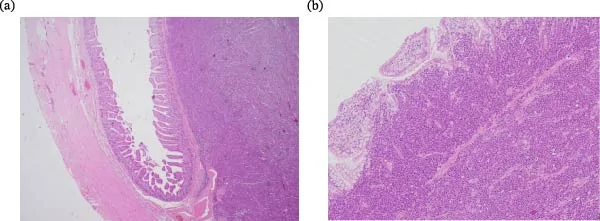
(a) A 20× H&E section of small bowel showing a mass composed of atypical plasma cells, present in the muscularis propria and expanding the submucosa. (b) A 100× H&E microscopic image demonstrating atypical plasmocytic infiltrate extending to the submucosa and mucosa.

**Figure 8:**
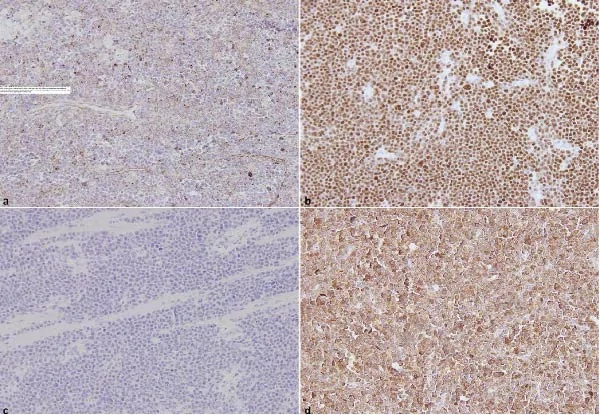
(a) A 200× CD138 IHC stain showing weak but positive expression throughout the atypical plasmocytic population. (b) A 200× MUM1 IHC stain showing retained strong expression. (c) and (d) 200x kappa and lambda light chain IHC stains, respectively, showing diffuse positivity for lambda light chain, with negativity for kappa light chain, consistent with lambda light chain restriction in the atypical plasma cell population.

Gross examination of the resected specimen revealed a 40 mm × 30 mm × 15 mm intraluminal, well‐circumscribed nodule with associated serosal changes (Figures 5 and 6). Histopathological analysis demonstrated a mass composed of atypical large plasmacytoid cells with abundant mitotic figures, expanding the submucosa with extension into the overlying mucosa and with involvement of the muscularis propria and serosal surface (Figures 7 and 8). Immunohistochemistry demonstrated weakly positive CD138 staining and strong positivity for MUM1 with lambda light chain restriction. Fluorescence in situ hybridisation (FISH) analysis was performed; no translocations were identified for CCND1 (cyclin D1) or IGH/MYC (associated with Burkitt‐type rearrangements). No rearrangements were detected for BCL2, IGH/MYC or BCL6. These findings were consistent with extramedullary myeloma with anaplastic features. Resection margins were clear, and one regional lymph node showed tumour involvement.

Post‐operative management followed a coordinated multidisciplinary approach. The haematology team managed the systemic myeloma relapse with corticosteroids and arranged systemic therapy following discharge. The endocrinology team supervised appropriate steroid tapering. The patient tolerated early oral intake and passed flatus by postoperative day 2, with restoration of intestinal function by day 5. She was discharged on day 6 on oral corticosteroids with scheduled haematology follow‐up.

## 3. Discussion

Intussusception in adults is uncommon and is often indicative of an underlying pathology, with malignancies accounting for a significant proportion of cases. In a review of adult intussusception cases, approximately 65%–70% were associated with tumours, with malignant lesions comprising 30%–50% [10, 11]. In the context of MM, gastrointestinal involvement is rare, occurring in approximately 0.9% of cases, and is often associated with a poor prognosis [12, 13].

The clinical presentation of intussusception is often non‐specific, making preoperative diagnosis challenging. Common symptoms include intermittent abdominal pain, nausea, vomiting and signs of bowel obstruction [4]. Imaging studies, particularly contrast‐enhanced CT, are crucial for diagnosing intussusception and identifying the underlying cause [14, 15]. Prompt access to out‐of‐hours CT imaging is now integral to timely emergency general surgery decision‐making [15]. The characteristic ‘target’ or ‘sausage‐shaped’ appearance on CT is indicative of intussusception [16]. In this patient, CT imaging demonstrated jejunal intussusception with proximal bowel dilatation, enabling early surgical consultation.

The management of adult intussusception typically involves surgical intervention, given the high likelihood of an underlying pathological lead point. Intraoperative findings often dictate the extent of resection required. In this case, segmental resection of the involved bowel with primary anastomosis was performed. Alternative surgical options for obstructive intussusception include stoma formation or intestinal bypass. For this patient, the proximal location of the lesion, active malignancy and recent high‐dose corticosteroid use were all considered in the decision between primary anastomosis and stoma formation. A bypass procedure may be considered in palliative patients with disseminated peritoneal disease, limited bowel mobility or stoma aversion. This patient’s clinical stability, ASA grade and body habitus permitted a laparoscopic approach with a targeted periumbilical incision.

The role of corticosteroids in this case warrants a specific discussion. High‐dose dexamethasone was administered preoperatively under haematology guidance, with the rationale of reducing the tumour burden and peritumoral oedema to alleviate mechanical obstruction—a strategy supported in systemic MM [17]. However, the evidence for corticosteroids reducing the size of a discrete extramedullary plasmacytoma causing mechanical obstruction is limited, and in this case, the obstruction did not resolve despite 3 days of steroid therapy. This aligns with the emerging literature suggesting that extramedullary disease may behave more aggressively and respond less predictably to systemic agents than intramedullary disease. Beyond their oncological role, corticosteroids present additional surgical considerations: they can blunt systemic inflammatory responses, potentially masking classic signs of abdominal pathology such as peritonism or fever [18], and immunosuppression increases the risk of post‐operative infection and may impair anastomotic healing [17]. These factors reinforce the importance of early operative decision‐making rather than prolonged conservative trials in this patient population.

As the demographic of emergency general surgery continues to shift—with an increasing number of oncology patients presenting with complex comorbidities—clinicians must maintain a high index of suspicion and a lower threshold for imaging and intervention [19, 20]. A review of the literature, conducted through searches of PubMed, Embase and Web of Science, reveals that only a limited number of cases have documented small bowel intussusception secondary to extramedullary plasmacytoma. The earliest report was published in 1986 by Hill and Yudelman, [21], describing the first documented case of intussusception in a patient with MM. Subsequent reports have echoed this observation in both relapsed and active myeloma patients [22–27]. Across these cases, the consistent identification of a plasmacytoma as the pathological lead point reinforces the importance of considering this rare but significant aetiology in myeloma patients presenting with vague or non‐specific abdominal symptoms. With the advent of more effective chemotherapeutic agents, overall survival in MM has significantly improved; consequently, the incidence of delayed or atypical presentations—such as small bowel obstruction from secondary extramedullary plasmacytoma—is expected to rise. Recognition of these rare manifestations is becoming increasingly relevant to the general surgeon, who may be the first clinician to encounter such cases in the acute setting.

## 4. Conclusion

This case illustrates a rare but important cause of small bowel obstruction in patients with MM: intussusception secondary to an extramedullary plasmacytoma. A high index of suspicion for gastrointestinal extramedullary plasmacytoma should be maintained in patients with active or previously treated MM who present with abdominal symptoms. These lesions can manifest acutely and mimic other causes of bowel obstruction, often requiring surgical intervention for both diagnosis and treatment. While high‐dose corticosteroids are a reasonable initial oncological strategy, their limited efficacy in resolving mechanical obstruction from discrete extramedullary deposits and the surgical risks they carry should prompt early operative planning. Early evaluation with CT imaging plays a pivotal role in identifying intussusception and mass lesions. Given the potential for aggressive histopathological features and the prognostic implications of extramedullary disease, prompt multidisciplinary management is essential. Recognising this rare but serious complication can lead to timely intervention and improved clinical outcomes.

## Funding

No funding was received for this manuscript.

## Ethics Statement

Written informed consent was obtained from the patient for the publication of this case report and any accompanying images. Patient confidentiality has been maintained throughout.

## Conflicts of Interest

The authors declare no conflicts of interest.

## Data Availability

Data sharing is not applicable to this article as no datasets were generated or analysed during the current study.
